# A Bilayer 2D-WS_2_/Organic-Based Heterojunction for High-Performance Photodetectors

**DOI:** 10.3390/nano9091312

**Published:** 2019-09-13

**Authors:** Feng Huang, Jing Zhou Li, Zhu Hua Xu, Yuan Liu, Ri Peng Luo, Si Wei Zhang, Peng Bo Nie, Yan Fei Lv, Shi Xi Zhao, Wei Tao Su, Wen Di Li, Shi Chao Zhao, Guo Dan Wei, Hao Chung Kuo, Fei Yu Kang

**Affiliations:** 1 College of Materials & Environmental Engineering, Hangzhou Dianzi University, Hangzhou 310018, China; grafenggh@hdu.edu.cn (F.H.); zhuhuaxu@hdu.edu.cn (Z.H.X.); lvyanfei@hdu.edu.cn (Y.F.L.); suweitao@hdu.edu.cn (W.T.S.); 2 Tsinghua-Berkeley Shenzhen Institute (TBSI), Tsinghua University, Shenzhen 518055, China; lijingzhou1989@163.com (J.Z.L.); luorp19@mails.tsinghua.edu.cn (R.P.L.); zsw14@mails.tsinghua.edu.cn (S.W.Z.); nie_pb@163.com (P.B.N.); fykang@sz.tsinghua.edu.cn (F.Y.K.); 3 Tsinghua Shenzhen International Graduate School, Tsinghua University, Shenzhen 518000, China; liuyuan18@mails.tsinghua.edu.cn (Y.L.); zhaosx@sz.tsinghua.edu.cn (S.X.Z.); 4 Department of Mechanical Engineering, The University of Hong Kong, Pokfulam, Hong Kong, China; liwd@hku.hk; 5 Institute of Electro-Optical Engineering, National Chiao Tung University, Hsinchu 30010, Taiwan; hckuo@faculty.nctu.edu.tw; 6 Department of Electrical Engineering and Computer Sciences and Tsinghua-Berkeley Shenzhen Institute (TBSI), University of California at Berkeley, Berkeley, CA 94720, USA

**Keywords:** 2D-WS_2_, photodetector, organic semiconductor, responsivity

## Abstract

Two-dimensional (2D) tungsten disulfide (WS_2_) has inspired great efforts in optoelectronics, such as in solar cells, light-emitting diodes, and photodetectors. However, chemical vapor deposition (CVD) grown 2D WS_2_ domains with the coexistence of a discontinuous single layer and multilayers are still not suitable for the fabrication of photodetectors on a large scale. An emerging field in the integration of organic materials with 2D materials offers the advantages of molecular diversity and flexibility to provide an exciting aspect on high-performance device applications. Herein, we fabricated a photodetector based on a 2D-WS_2_/organic semiconductor materials (mixture of the (Poly-(*N*,*N*′-bis-4-butylphenyl-*N*,*N*′-bisphenyl) benzidine and Phenyl-C61-butyric acid methyl ester (Poly-TPD/PCBM)) heterojunction. The application of Poly-TPD/PCBM organic blend film enhanced light absorption, electrically connected the isolated WS_2_ domains, and promoted the separation of electron-hole pairs. The generated exciton could sufficiently diffuse to the interface of the WS_2_ and the organic blend layers for efficient charge separation, where Poly-TPD was favorable for hole carrier transport and PCBM for electron transport to their respective electrodes. We show that the photodetector exhibited high responsivity, detectivity, and an on/off ratio of 0.1 A/W, 1.1 × 10^11^ Jones, and 100, respectively. In addition, the photodetector showed a broad spectral response from 500 nm to 750 nm, with a peak external quantum efficiency (EQE) of 8%. Our work offers a facile solution-coating process combined with a CVD technique to prepare an inorganic/organic heterojunction photodetector with high performance on silicon substrate.

## 1. Introduction

Two-dimensional materials (2D), such as graphene, hexagonal boron nitride, transition metal dichalcogenides, tin sulfide, black phosphorus, ultrasmall bismuth quantum dots, and selenium nanoflakes, have become optically active semiconductors in biomedicine, ion detectors, and photodetectors (PDs). Photodetector-based graphene exhibits a large-response wavelength range and a high on/off ratio. Two-dimensional black phosphorus, with an anisotropic band structure, shows linear dichroism and anisotropic absorption. Photodetector-based monolayer black phosphorus exhibits excellent polarization sensitivity with a large bandwidth. Photoelectric properties of transition metal dichalcogenides (TMDs) have a remarkable change, as bulk transforms into a monolayer or multilayers, which have high optical absorption rates and suitable band gaps. Photodetector-based monolayer transition metal dichalcogenides (TMDs) show high quantum efficiency and low response times [1,2,3,4,5,6,7,8,9,10,11,12]. Among these 2D materials, tungsten disulfide (WS_2_) layers with high mobility of 1000 cm^2^·v^−1^·s^−1^, a high optical absorption coefficient of ca. 10^6^ cm^−1^, and a band gap of 1.9 eV [13,14,15,16] are typical n-type 2D materials for electronic and optoelectronic device applications, making them compatible to combine with other materials to construct 2D van der Waals heterostructures.

Organic materials are suitable materials to vertically stack together with 2D WS_2_ layers to form an energy-favorable inorganic/organic heterojunction, providing an effective interface to separate the electron-hole (e-h) pairs excited by incident photons. A significant advantage to employing organic materials is due to their facile solution processability [17,18,19,20,21,22]. Nowadays, it remains challenging to form uniform 2D WS_2_ thin films on a large scale due to the coexistence of discontinuous and isolated single layers and multilayers [23,24,25,26,27]. Due to the excellent flexibility of organic materials, the surface of the isolated 2D WS_2_ layers could be effectively smoothed out and interconnected together, filling pinholes and vacancies of the 2D layers underneath. Recently, research has been intensively carried out to combine 2D inorganic materials with organic materials to form energy-favorable junctions for efficient exciton dissociation and charge transport [28,29,30,31,32,33,34]. Two-dimensional CsPbBr_3_ nanosheets have been assembled into flexible heterojunction films with phenyl-C61-butyric acid methyl ester (PCBM), and the 2D CsPbBr_3_/PCBM photodetectors exhibited an enhanced responsivity of 10.85 AW^−1^ and an ultrahigh detectivity of 3.0 × 10^13^ Jones [30]. The van der Waals heterojunction formed between pentacene and 2D MoS_2_ has demonstrated an ultrafast charge transfer of 6.7 ps (especially the charge-separated state that lives for 5.1 ns) up to an order of magnitude longer than the pure 2D heterojunctions, suggesting the benefits of junction-stacking organic/2D materials [31]. Therefore, an emerging field in the integration of organic materials with 2D materials provides an exciting aspect on continuous 2D device fabrication. A hole transport material, Poly-(*N,N*′-bis-4-butylphenyl-*N,N*′-bisphenyl) benzidine (Poly-TPD), and an electron transport material, PCBM, are often used for hole and electron transporting layers in organic photodetectors and solar cells [35,36,37]. Two-dimensional WS_2_ (conductive band edge at ca. −3.84 eV and valence band edge at ca. −5.82 eV for a monolayer) is a perfect match for the Poly-TPD/PCBM mixture (LUMO at −4.1 eV of PCBM to accept electrons and HOMO at −5.2 eV of Poly-TPD to accept holes), which is favorable for exciton dissociation [38,39,40,41,42,43].

The growth of monolayer WS_2_ is always a big challenge, especially for the large-scale monolayer WS_2_. Herein, the large-scale monolayer WS_2_ was successfully prepared using ZnO film as an auxiliary material on an SiO_2_/Si substrate through chemical vapor deposition (CVD) for the first time. With a convenient solution spin-coating technique, the blend film of mixed Poly-TPD and PCBM at a weight ratio of 1:1 was deposited on top of the 2D WS_2_ to form an ordered bilayer inorganic/organic diode. The 2D WS_2_ thin films, obtained from ZnO-controlled growth through chemical vapor deposition (CVD), provided inert and solid surfaces. The van der Waals force interactions of 2D WS_2_ and organic layers could allow for the planar growth of organic-based films with larger crystal grain sizes. In the meantime, organic layers could smooth out the surface of the 2D WS_2_ layer for continuous device fabrication, which could substantially suppress dark currents. Thus, functionalized organic thin films and suitable architectures with 2D WS_2_ must be well optimized in order to realize inorganic/organic-based PDs with high performance.

Overall, the 2D WS_2_/Poly-TPD/PCBM PD obtained a broad spectral response from 500 nm to 750 nm with a peak external quantum efficiency (EQE) of 8% at a wavelength of 527 nm. When illuminated with a 450-nm excitation laser at a power intensity of 0.14 mW/cm^2^, the PD showed a gate-tunable responsivity, a specific detectivity (D*), and an on/off ratio of 0.1 A/W, 1.1 × 10^11^ Jones, and 100, respectively. Meanwhile, the responsivity dramatically increased with the laser excitation intensity, then saturated to 17 A/W when the drain voltage *V_D_* was 10 V. As a result, the 2D inorganic/organic bilayer heterojunction was successfully demonstrated for a high-performance PD and could extend to other organics and 2D materials. 

## 2. Materials and Methods

### Fabrication and Characterizations

**Figure 1:**
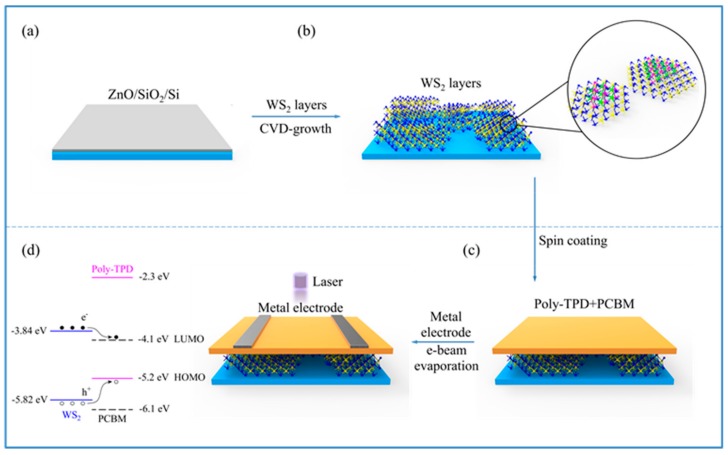
Illustration (color online) of the fabrication procedure of the photodetector based on the organic semiconductor/inorganic tungsten disulfide (WS_2_) heterojunction. (**a**) The silicon substrate with ZnO and SiO_2_ film (ZnO/SiO_2_/Si). (**b**) Two-dimensional (2D) WS_2_ growth on the surface of SiO_2_/Si through a chemical vapor deposition (CVD) method. During the growth of the WS_2_, ZnO was removed from the SiO_2_/Si. (**c**) (Poly-(*N*,*N*′-bis-4-butylphenyl-*N*,*N*′-bisphenyl) benzidine and Phenyl-C61-butyric acid methyl ester (Poly-TPD/PCBM) (1:1) organic film preparation by spin-coating on the surface of the 2D WS_2_. (**d**) Metal electrode evaporation on the surface of the Poly-TPD/PCBM film.

ZnO film was prepared on an SiO_2_/Si substrate at a thickness of ca. 100 nm through DC reactive magnetron sputtering (Figure 1a) [44]. Metal zinc (DingWei, Dongguan, China) was used as a target. Argon (Ar) was used as the sputtering gas and oxygen as the reactant. Ar/O_2_ (Ar 20 sccm, O_2_ 60 sccm, 0.3 Pa) was introduced into the sputtering chamber. A negative bias voltage of −300 V was applied to keep glow discharge for 30 min. WS_2_ film was prepared through a CVD method (Figure 1b) [45,46]. The WS_2_ powders (Aladdin, Shanghai, China) were loaded in the center of the CVD system and heated to 1000 °C from room temperature over 30 min. An SiO_2_/Si substrate with a layer of ZnO (ZnO/SiO_2_/Si) was placed in the downstream region of the CVD. During the above process, the substrate was heated to ca. 700 °C, which was kept for 60 min. Ar/H_2_ (H_2_ 5%, 105 Pa, 35 sccm) was used as a carrier gas [47]. The spin-coating method was applied to prepare the Poly-TPD/PCBM (1:1) thin film on the top of the WS_2_ thin film (Figure 1c). The Poly-TPD (Aladdin, Shanghai, China) and PCBM (Aladdin, Shanghai, China) were first dissolved in chlorobenzene solvent with a mass concentration of 10 mg/mL. The spin-casting processes were performed in a glove box. The blend layer of organic materials was spin-coated at 3000 rpm for 50 s, followed by annealing at 110 °C for 30 min. Cr–Au electrodes were e-beam-evaporated on top of organic thin films through a shadow mask with an exposed active area under a vacuum of 1.3 × 10^−3^ Pa (Figure 1d). Here, the channel width of the device was 20 μm, and the length was 100 μm. The final structure of the typical device, on top of an oxidized silicon wafer, was Au/Poly-TPD/PCBM/WS_2_/Au. The doped silicon could be used as a back gate and SiO_2_ as a gate dielectric. A schematic diagram of the prepared 2D WS_2_/organic photodetector with the respective energy level alignments is shown in Figure 1d.

X-ray diffraction (XRD) was performed on a Thermo ARLXTRA (Geneva, Switzerland). X-ray photoelectron spectroscopy (XPS) spectra were performed on an Ulvac-Phi PHI5000 Versaprobe II (Kanagawa, Japan). UV-Vis absorption was performed on a Shimadzu UV-3600 (Kyoto, Japan). Field emission scanning electron microscopy (FESEM) was performed on a FEI Apreo S HiVac (Hillsboro, OR, USA). Raman spectra and photoluminescence (PL) were performed on a micro-Raman setup consisting of a 532-nm solid state laser, a Nikon inverted microscope (Ti eclipse, Tokyo, Japan), a long-pass edge filter (Semrock, New York, NY, USA), and a Raman spectrometer (Horiba, iHR320, Kyoto, Japan). An optical microscope image and a luminescence image were taken on a Jiangnan MV 3000 digital microscope (Nanjing, China). An electrical measurement was carried out on an Agilent 4200 SCS (CA, America) and a LakeShore TTPX (Columbus, OH, USA). The photodetection properties were examined with laser excitation wavelengths of 450 nm to 750 nm.

## 3. Results and Discussion

**Figure 2:**
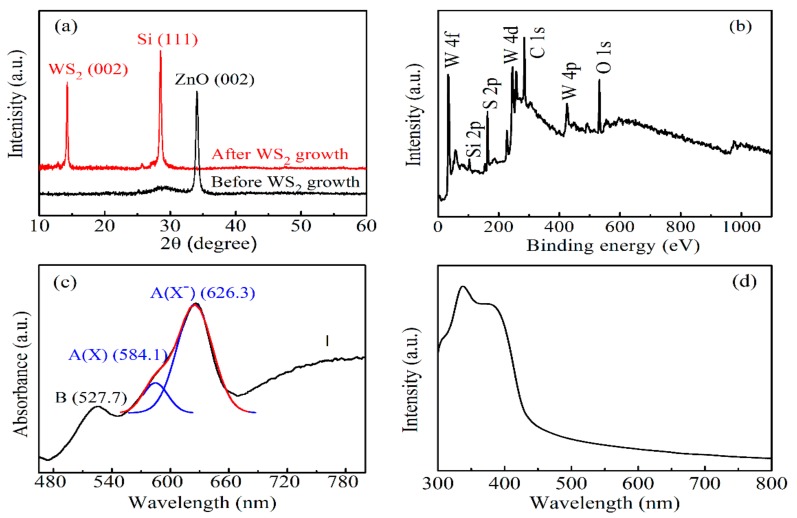
(**a**) The X-ray diffraction (XRD) spectra of the ZnO/SiO_2_/Si substrate (black line) and WS_2_ film (red line). (**b**) X-ray photoelectron spectroscopy (XPS) spectrum of the WS_2_ film. UV-Vis absorption spectrum of the (**c**) WS_2_ film and (**d**) Poly-TPD/PCBM (1:1).

Figure 2a shows the XRD spectra of the ZnO/SiO_2_/Si substrate before and after the growth of the WS_2_ film. The XRD data reveal that WS_2_ film along with (002) direction was prepared on the Si substrate [48]. The full width at half maximum (FWHM) of the WS_2_ XRD peak (002) was similar to that of the Si single crystal, indicating that a WS_2_ film with a single crystalline with a large crystal grain size was obtained. There was no ZnO XRD peak that existed after complete 2D WS_2_ growth (Figure 2a) [34], which was further confirmed by the XPS spectrum, since no Zn^2+^ signal (Zn 2P_1/2_ at 1021.75 eV and Zn 2P_3/2_ at 1044.7 eV) was detected (see Figure 2b) [49]. The detailed mechanism of 2D WS_2_ controlled growth was discussed in our previous work [50]. The shape and scale of the monolayer WS_2_ was affected by the atomic ratio of *W/S*. When the atomic ratio of W/S was less or larger than 1:2, the monolayer WS_2_ would only grow into a small triangular shape. Therefore, it was critical to maintain the atomic ratio of W/S to as close to 1:2 as possible. Since ZnO whiskers can absorb extra W atoms to form a ZnWO_4_ compound in a way that adjusts the atomic ratio of W/S back to 1:2, the WS_2_ could be promoted further to grow into large-scale monolayer domains. We speculated the ZnWO_4_ was sublimated and removed from the substrate during the subsequent growth stage.

The UV-Vis absorption spectrum of the WS_2_ film is shown in Figure 2c. The A peak (located at 575–670 nm) and B peak (located at 527.7 nm) were due to the A and B exciton absorptions, respectively [51,52]. Due to spin-orbit coupling, the valence band split into two sub-bands (*v*_1_ and *v*_2_, *v*_1_ < *v*_2_) at the *K* point of the Brillouin zone. Transitions from *v*_1_ and *v*_2_ to the minimum of the conduction band corresponded to the B exciton and A exciton, respectively [53]. The A peak consisted of two peaks at 584.1 nm (A(X)) and 626.3 nm (A(X^−^)). A(X) was due to the neutral exciton absorption, and A(X^−^) was due to the charged exciton absorption [54]. In addition, there was a broad peak I at the low-energy side of the A peak (>700 nm). The weak and broad peak I was ascribed to the indirect band gap transition of the multilayers/bulk [24]. Figure 2d shows the absorption spectrum of the organic Poly-TPD/PCBM (1:1) thin films, with two strong absorption peaks in the near-UV region (300–420 nm).

**Figure 3:**
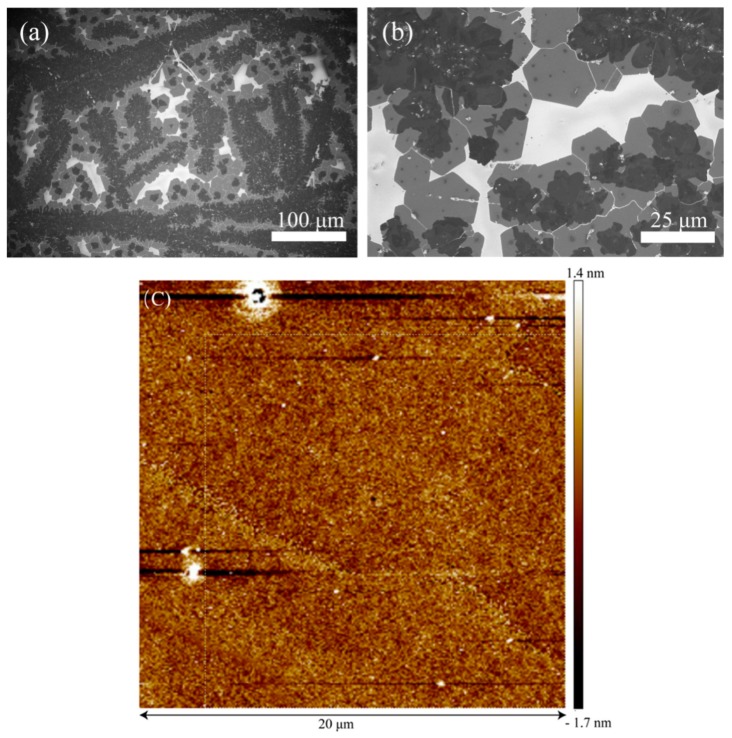
(**a**,**b**) Typical field emission scanning electron microscopy (FESEM) images of the WS_2_ film. Images (a,b) were taken at different locations. (**c**) Atomic force microscope (AFM) image of 1:1 Poly-TPD and PCBM mixture.

Figure 3 shows typical FESEM images of WS_2_ film grown on the silicon substrate via the CVD method. The white-colored area in Figure 3a,b is the SiO_2_/Si substrate. The gray-colored areas with the hexagonal shape are WS_2_ monolayers. The hexagon morphology indicated that the WS_2_ monolayer we prepared had a single crystalline with a large crystal grain size. The dark-colored areas with irregular morphology were due to the WS_2_ multilayers. The small-sized multilayers were stacked on a monolayer with a large domain size. During WS_2_ growth, hexagonal WS_2_ monolayers merged together. As shown in Figure 3b, cracks in these hexagonal interfaces could be clearly viewed, and they could have formed during the CVD growth or cooling process. For the Poly-TPD/PCBM (1:1) blend films, the roughness was 0.5 nm in Figure 3c, indicating that Poly-TPD and PCBM were uniformly mixed together, which was required for the subsequent Au electrode deposition. If the surface of the organic blend film were not smooth, the deposited Au atoms could penetrate through the vacancies or voids of the 2D WS_2_ materials, and the as-prepared devices would be very leaky. Therefore, the uniform feature of the blend film was crucial to a successful 2D WS_2_/organic photodetector fabrication.

**Figure 4:**
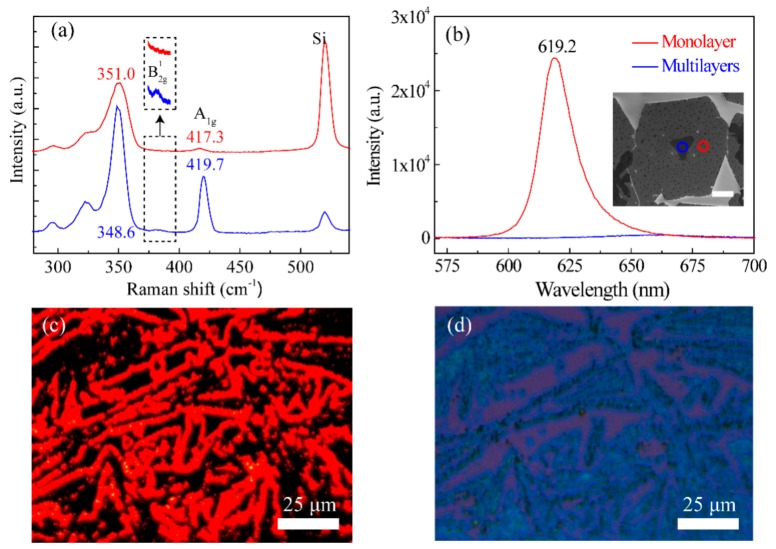
The Raman (**a**) and photoluminescence (PL) spectra (**b**) of the WS_2_ monolayer (red line) and multilayers (blue line). Inset in (a) is a detailed illustration of the *B_2g_^1^* and *A_1g_* modes. The insert in (b) is an SEM image, and the scale bar represents 5 μm. (**c**) Photoluminescence (PL) and (**d**) optical microscopy images of WS_2_ mono-/multimixed layers taken at the same location.

Typical Raman spectra of the WS_2_ films at room temperature with a 532-nm laser excitation are shown in Figure 4a. The red and blue curves in Figure 4a approximately correspond to the areas circled with a red line and blue line in the inset of Figure 4b, respectively. The Raman peak of silicon (Si) at 520 cm^−1^ was used to calibrate the Raman spectra of the as-prepared WS_2_ thin films. Two typical Raman peaks at 351 cm^−1^ (*E_2g_*^1^) and 417.3 cm^−1^ (*A_1g_*) were observed in the red curve [55]. The frequency difference between the two modes was 66.3 cm^−1^. The peak intensity ratio of *A_1g_/E_2g_^1^* was 0.029. The narrow frequency difference, small peak intensity ratio, and weak *A_1g_* intensity indicated that the WS_2_ domain labeled with a red circle in Figure 4c was confirmed to be a monolayer [56]. For the blue curve in Figure 4a, the *E_2g_^1^* redshifted to 348.6 cm^−1^, and the *A_1g_* blueshifted to 419.7 cm^−1^. The frequency difference increased to 71.1 cm^−1^. In addition, the intensity ratio increased to 0.25, indicating that the WS_2_ domain circled with a blue line in the inset of Figure 4b had a multilayer feature [24]. Besides the *E_2g_^1^* and *A_1g_* peaks, we found a *B_2g_^1^* peak as well in the blue curve. The phonon mode *B_2g_^1^* was only active in the multilayers [57,58]. Thus, the WS_2_ domain corresponding to the blue curve had multilayers. In addition, the absence of the B_2g_^1^ peak in the red curve indicates that the corresponding WS_2_ domain was a monolayer. Figure 4b shows the typical photoluminescence (PL) spectra of the WS_2_ monolayer (red line) and multilayers (blue line) with a 532-nm laser excitation. Interestingly, the multilayer WS_2_ showed a faint PL emission without obvious peaks, since multilayer WS_2_ had indirect band gap characteristics. In contrast, monolayer WS_2_ exhibited an intense and sharp PL spectrum with a typical peak at 619.2 nm, which indicated a direct band gap of the WS_2_ monolayer [59]. Correspondingly, the PL mapping (Figure 4c) of the in situ optical microscopic view in Figure 4d clearly shows the monolayer WS_2_ appearing as red lines cross-linking with the multilayer WS_2_ (the dark region). Thus, the interconnected lines of the monolayer WS_2_ contributed to the continuity of the as-prepared WS_2_ films from the CVD method, which was consistent with the interconnected monolayers, as shown in the SEM images of Figure 3.

### Opto-Electronic Properties of Photodetector

**Figure 5:**
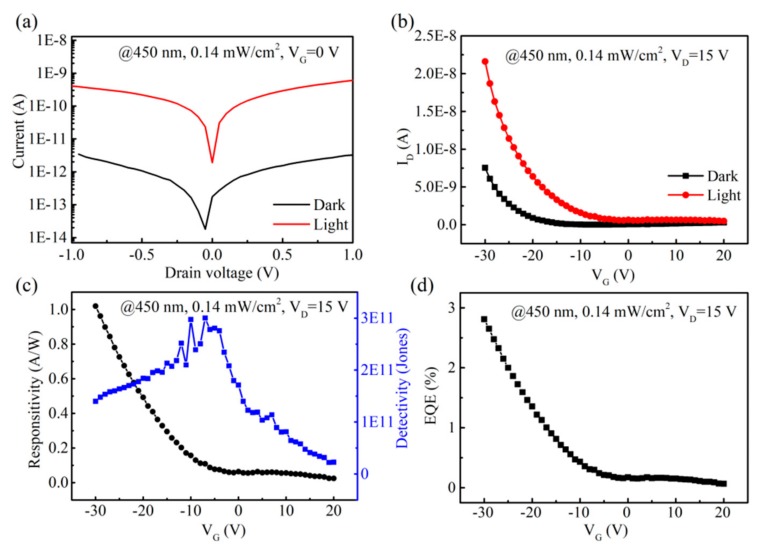
Performance of the WS_2_/Poly-TPD/PCBM-based photodetector. (**a**) Source-drain current (*I_D_*) versus drain voltage (*V_D_*). (**b**) *I_D_* versus gate voltage (*V_G_*) curves of the photodetector in dark (black line) and under laser (red line). (**c**,**d**) Responsivity and detectivity curves and the external quantum efficiency (EQE) of the device as a function of *V_G_* ranging from −30 V to 20 V. Measurements were performed at room temperature with a laser at 450 nm. The light intensity was 0.14 mW/cm^2^. The *V_G_* was 0 V in (a), and the *V_D_* was 15 V in (b–d).

The linear scale of the *I*-*V* characteristics of the bilayer photodetectors was measured in the dark and under light with an intensity of 0.14 mW/cm^2^ and a laser excitation wavelength of 450 nm (Figure 5a). The channel width of the device was 50 μm, and the length was 200 μm. The *I*–*V* curves showed a linear and symmetrical feature when no gate voltage was applied (*V_G_* = 0 V), which confirmed that the as-fabricated device had ohmic contacts between the blend film and the Cr–Au electrodes. Figure 5b shows that the photocurrent increased quickly, with the gate voltage (*V_G_*) sweeping from −30 V to 20 V. The responsivity (*R*) and detectivity (D*) of the aforementioned photodetectors under illumination were calculated according to

(1) $$R=\frac{I_{light}-I_{dark}}{PA},$$

(2) $$D^{*}=\frac{R}{{\left(2e\frac{I_{dark}}{A}\right)}^{1/2}},$$

where *I*_light_ is the photocurrent, *I_dark_* is the dark current, *P* is the light power density, *A* is the effective area of the photodetector, and *e* is the electronic charge. Figure 5c plots responsivity and detectivity, which increased with applied negative *V_G_*. On the one hand, these indices rose as the applied voltage increased, and they grew linearly when the voltage was lower than −10 V, which suggests that not only was the photocurrent far larger than the dark current (Figure 5c), but also that a quite low power (voltage) input was required to amplify the photocurrent to the highest order of magnitude. With a −30 V bias voltage, the photocurrent (*I_light_*) and dark current (*I_dark_*) were 2.16 × 10^−8^ A and 7.56 × 10^−9^ A, respectively. Therefore, the as-fabricated 2D WS_2_/Poly-TPD/PCBM/Au photodetector achieved an *R* of 1.01 A/W and a high D* of 1.4 × 10^11^ Jones at the 450-nm laser excitation (*V_G_* = −30 V). The external quantum efficiency (EQE) was obtained by

(3) $$EQE=\frac{Rhc}{e\lambda }$$

where hc/λ is the photon energy. The EQE (Figure 5d) exhibited the same gate voltage dependence trend as *R*, reaching about 3% at *V_G_* = −30 V. As can be seen in Table 1, the obtained values in this work were comparable to those obtained in other work. Compared to WS_2_ devices without organic materials [60,61], our device exhibited better performance, which indicated that the organic materials could improve the 2D material photodetector performance.

**Figure 6:**
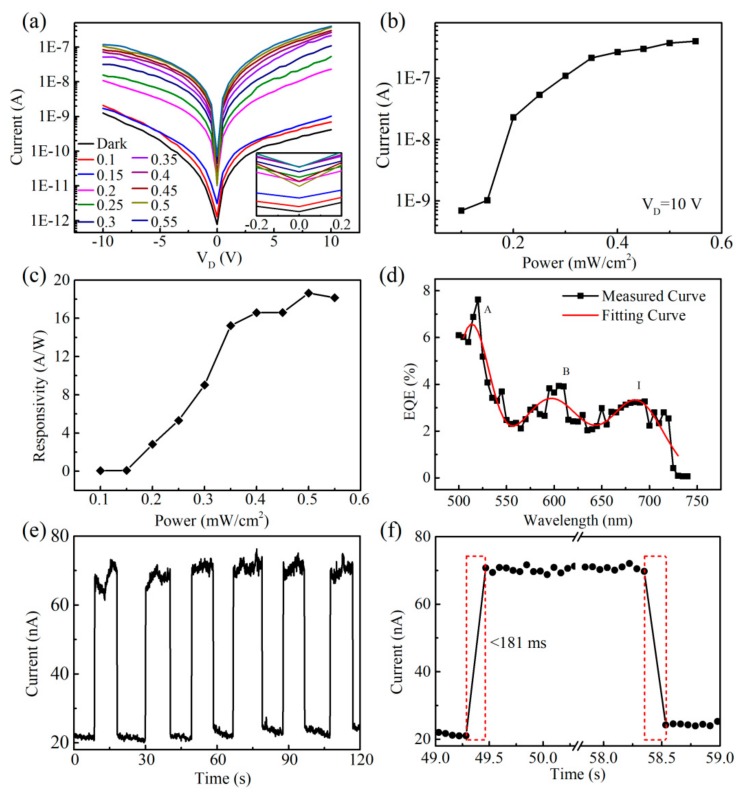
(**a**) The photocurrent versus applied *V_D_* sweeping from −10 V to 10 V (*V_G_* = 0 V) at different light intensities. The inset shows the detailed *I*–*V* characteristics in the 0 V region. (**b**) The dependence of the photocurrent (*I_D_,_light_*) (in (a)) on the laser intensity (mW/cm^2^) (*V_G_* = 0 V, *V_D_* = 10 V). (**c**) Power intensity dependence of the responsivity (*V_G_* = 0 V, *V_D_* = 10 V). (**d**) Spectral dependence of the EQE using a 100-ps pulse laser (*V_G_* = 0 V, *V_D_* = 10 V). (**e**) Photoresponse in the dark and under 450-nm laser irradiation with a light intensity of 0.14 mW/cm^2^ (*V_G_* = 0 V, *V_D_* = 10 V). (**f**) Rise and decay time of the photodetector (*V_G_* = 0 V, *V_D_* = 10 V).

Figure 6a shows the photocurrent versus applied *V_D_* sweeping from −10 V to 10 V. In addition, 10 different illumination intensities were applied to the WS_2_/organic device with varied laser intensities ranging from 0.1 mW/cm^2^ to 0.55 mW/cm^2^. These *I*–*V* plots showed a linear increase of the photocurrent with the applied voltage. In Figure 6b, the dependence of the photocurrent (*I_D_,_light_*) (in Figure 6a) on the laser intensity (mW/cm^2^) was plotted on a log scale (*V_D_* = 10 V). The *I_D_,_light_* increased gradually with power intensity when the power was 0.4 mW/cm^2^ and then showed a saturating tendency with higher power intensity. As shown in Figure 6c, the responsivity *R* increased nearly linearly to 17 A/W with incident laser power when the power intensity reached 0.4 mW/cm^2^, and then it was gradually saturated with further increased power intensity (*V_D_* = 10 V).

The broad-spectrum response of the as-prepared bilayer photodetector was investigated with an incident laser with a wavelength varying from 500 nm to 750 nm (Figure 6d). Herein, the EQE was calculated from the *I*–*V* curves (*V_D_* = 10 V and *V_G_* = 0 V), and the incident light was a 100-ps pulse laser with a 4-MHz frequency. The peaks in Figure 6d corresponded well to the A exciton (λ = 626 nm), B exciton (λ = 527 nm), and indirect band gap (I) absorption, as shown in Figure 2c, indicating that the effective absorption in the WS_2_ thin films actively contributed to the photocurrent generation [24,51,52]. As shown in Figure 1d, the LUMO at −4.2 eV of PCBM and the HOMO at −5.2 eV of Poly-TPD were well aligned with the conduction and valence levels of WS_2_ to dissociate the excitons typically generated inside the WS_2_ films, with an exciton binding energy of 0.3 eV to 0.7 eV [15,16]. The reliable and rapid response speed of the bilayer photodetector was examined with pulsed laser illumination, as shown in Figure 6e,f. The PD promptly responded to 450 nm of light at the millisecond level (<181 ms), giving rise to a sharply enhanced and decayed photocurrent upon multiple switching cycles.

The 2D WS_2_/Poly-TPD/PCBM bilayer photodetectors with few layers of WS_2_ could detect different photon wavelengths and a wide range of incident intensities, making it a strong candidate for constructing novel optoelectronic devices. We attribute the excellent photoresponse to the appropriate band gap, the high quality of the CVD-grown single/multilayer WS_2_, and the energy-favorable heterojunction. The as-prepared WS_2_ had excellent photon absorption throughout a wide range of 500 nm to 750 nm, allowing enough exciton (or electron-hole pair) generation. As shown in Figure 1d, 2D WS_2_ (conductive band edge at ca. −3.84 eV and valence band edge at ca. −5.82 eV for the monolayer) was a perfect match with the Poly-TPD/PCBM mixture (LUMO at −4.1 eV of PCBM to accept electrons and HOMO at −5.2 eV of Poly-TPD to accept holes). The energy offset of the WS_2_ and organic blend film (the offset between WS_2_ and Poly-TPD was 0.62 eV and between WS_2_ and PCBM was 0.26 eV) was sufficiently high enough to dissociate excitons generated from monolayer WS_2_, since the typical exciton binding energy was around 0.71 ± 0.01 eV [15]. Therefore, Poly-TPD was favorable for hole transport and PCBM for electron transport to their respective electrodes. Notably, Poly-TPD and PCBM were intermixed throughout the entire organic blend film, and it is highly possible that portions of the electron and hole carriers recombined first before arriving at their electrodes, resulting in a relatively low EQE when the *V_G_* = 0 V (Figure 5d). Therefore, the built-in electric field needed to be applied through the gate voltage, and more photogenerated electrons and holes could quickly drift away to their electrodes. As shown in Figure 5d, with the increase in the gate voltage applied, the current increased exponentially, which is typical diode behavior. As a result, the EQE could be modulated to 3% at *V_G_* = −30 V.

Herein, the limited trap state in the CVD-grown WS_2_ layer also greatly reduced the exciton quenching, and photoconductivity might have dominated the fast response time, which was consistent with the nearly linear dependence of the photocurrent on incident laser power intensity (Figure 6b). However, photocurrents higher than 0.1 uA were gradually saturated with further increased laser power intensity, indicating that the charge collection efficiency caused a limiting factor due to relatively low carrier mobility in organic blend films. Correspondingly, chances for electron and hole-free carriers under higher irradiation excitations to recombine increased before collection by their respective Au electrodes. Notably, this saturated photocurrent behavior was quite different from pure 2D WS_2_-sandwiched PDs, which typically have nonlinear dependence on the incident laser power intensity P^0.5^ [65]. As a result, the responsivity of the as-prepared bilayer 2D-WS_2_/organic photodetector had a saturated trend quite similar with the radiant power intensity, reaching a maximum of 17 A/W. The wide visible spectrum response (Figure 6d) clearly showed that single/multilayers of WS_2_ laminated with organic blend films is a promising device architecture for high-performance optoelectronic applications. Optimization of the organic blend layer conductivity will further improve this unique 2D/organic bilayer photodetector performance. 

## 4. Conclusions

In summary, we prepared an inorganic/organic heterojunction photodetector through highly oriented 2D WS_2_ film and Poly-TPD/PCBM blend organic films. The results revealed that the photodetector had high responsivity and detectivity at room temperature, exhibiting a high detectivity of 1.1 × 10^11^ Jones at zero-gate voltage and a responsivity of 17 A/W. The spin-coated organic films effectively smoothed out the WS_2_ films, forming an energy barrier to significantly suppress the dark current. The on/off ratio of these bilayer PDs was as high as 100, and a rise time of less than 181 ms was obtained, indicating fast electron/hole dissociation at the interface of the 2D WS_2_ and Poly-TPD/PCBM (1:1) organic layers. We attribute the excellent photoresponse to the appropriate band gap, the high quality of the CVD-grown single/multilayer WS_2_, and the energy-favorable heterojunction for efficient exciton separation. Further improvement on carrier mobility in organic layers will enhance the charge collection efficiency. Our work offers a solution-coating process combined with a CVD technique that can create high-crystalline 2D films for high-performance photodetectors over regular silicon substrates. The inorganic 2D/organic heterostructures formed will create a new and fruitful paradigm in optoelectronics.

## Figures and Tables

**Table 1:** Photoresponse parameters of different materials and devices.

Device Structure	Incident Light	*R* (A/W)	*D* (Jones)	Response Time	Ref.
2D WS_2_/Poly-TPD/PCBM/Au	450 nm	1.02	1.4 × 10^11^	<181 ms	This work
Monolayer WS_2/_Au	532 nm	0.59	6.5 × 10^10^	280 ms	[60]
Multilayer WS_2_/Au	635 nm	0.7	2.7 × 10^10^	4.1 s	[61]
MoTe_2_/Au	532 nm	0.0004	1.08 × 10^8^	42.5 μs	[62]
GeSe_2_/Au	450 nm	2.5	N/A	0.2 s	[63]
GeP/Au	532 nm	3.1	N/A	>1s	[64]
